# Perspective of an Advocate - political advocacy in African cancer dialogue

**DOI:** 10.1186/1750-9378-8-S1-S2

**Published:** 2013-07-15

**Authors:** Kwanele Asante-Shongwe

**Affiliations:** 1 BreastSens, Johannesburg, South Africa and AORTIC African Cancer Advocates Consortium (ACAC), South Africa

## Abstract

The burden of cancer is climbing in all of Africa, yet the continent’s healthcare and political systems have not prioritized cancer control and treatment-care. Sub-Saharan Africa is predicted to have a greater than 85% increase in the burden of cancer by 2030. However, African communities have little or no knowledge of cancer. As a result, many patients present with advanced disease at first consultation leading to poor outcomes. A focused approach needs to be adopted to address this growing public health threat. Robust engagement by patients and persons affected by cancer is needed to exert pressure on key public healthcare influencers especially, clinicians, researchers, political leaders and public health policy-makers to prioritize the disease and to ease the massive human suffering caused by cancer morbidities and mortalities.

## Introduction

Involving well-trained patient advocates in setting the research and overall cancer control agendas has been proven beneficial in countries such as the United States in improving the quality of care people with cancer receive. In addition, the involvement of patient advocates ensures that financial and other resources are spent where they are mostly needed rather than on studies or programs that are of academic interest but of little value in furthering medical understanding of the causes of cancer. It is imperative that research funds are spent on studies, which will lead to the development of preventative vaccines and treatments to eradicate cancer.

## Understanding patient advocacy

Patient advocacy is an area of lay specialization in healthcare concerned with patient education about health (rights) and how to obtain needed cancer treatment and control. Patient advocates provide the healthcare knowledge to the public at large and mainly offer their services on a non-profit basis. More broadly, patient advocacy can include groups that develop policies and legislation to improve systems or processes for patients [1]. Patient advocates can also be non-patients from disciplines like social-work or health insurance employed to assist patients unravel health plans and or to chaperone them through healthcare systems [1]. It is worth noting that working within the for-profit sector as a patient advocate may create a conflict of interest for an advocate as it makes it hard for them to protect patients’ interests within a sector that they are employed.

The discipline of patient advocacy is well-established in countries like the United States and the United Kingdom, where non-profit organizations (NGOs) are recognized as key healthcare partners with clinicians, healthcare policy-makers, political leaders and researchers. However, patient advocacy is still in its infancy in most African countries, especially in the area of cancer advocacy.

African healthcare practitioners and policy-makers still tend to practice paternalistic medicine, with little meaningful patient participation in treatment decision-making. Low literacy levels, lack of easy access to the internet, patriarchy and gender inequity in most African communities are responsible for the continuation of these unequal relations between physicians, policy makers and patients. A new paradigm of patient inclusion in all key healthcare decisions has to be created and a strong patient advocacy movement will be critical in ushering in a more egalitarian healthcare approach in Africa.

## Understanding political advocacy

Political advocacy is a process by an individual or group which aims to influence public policy and resource allocation decisions within political, economic, and social systems and institutions. Advocacy can include many activities that a person or organization undertakes including media campaigns, public speaking, commissioning and publishing research. Lobbying (often by interest groups like people with cancer) is a form of advocacy where a direct approach is made to legislators on an issue which plays a significant role in modern politics – for example access to equitable and evidence based healthcare for all citizens [1].

## The rising burden of cancer in Africa: Lessons from HIV/AIDS

Sub-Saharan Africa has a disproportionate burden of disease and faces a major public-health challenge from both communicable and non-communicable diseases [2]. The World Health Organization (WHO) estimates that about 551,200 cases of cancer were diagnosed in sub-Saharan Africa in 2008, with about 421,000 deaths [2]. Competing burden of disease from health issues such as tuberculosis, malaria and HIV/AIDS adversely impact the attention given to cancer at both political and public healthcare planning levels by African countries [2]. Sparse oncology equipment and infrastructure, poor supply of essential drugs, scarcity of qualified oncology specialists, inadequate hospital palliation and home based care services and poor cancer registries are the norm in most African countries to the detriment of patient care. The current lack of decisive political responses to cancer control care in Africa has echoes of a similar lack of focus initially accorded the HIV/AIDS pandemic by governments on the continent in the 1990s. Patients suffered and hundreds of thousands of lives were reported to have been lost as African leaders engaged in a non-scientific debate on whether HIV caused AIDS, refuting affirmative scientific evidence.

*The case of South Africa and HIV/AIDS: Africa and the global community were astonished when South Africa’s President Thabo Mbeki accused the West of impugning Africans’ sexual mores by suggesting that there was a scientifically proven causal sexual link between the human immune-deficiency virus and AIDS. Mbeki noted*, *“Convinced that* (*Africans*) *are but natural born*, *promiscuous carriers of germs*, *unique in the world*, *they* (*scientists*) *proclaim that our continent is doomed to an inevitable mortal end because of our unconquerable devotion to sin and lust.” *[3]*. In a study published in 2008*, *Harvard public health researchers argued that the “South African president’s refusal to accept medical evidence of the virus was a major obstacle to providing medicine to patients who desperately needed it.” *[4]*. The authors asserted that “Mbeki fought against scientific consensus that AIDS was caused by a viral infection that could be fought – though not cured – by sophisticated and expensive drugs.” *[4]*. It is estimated that more than 330*,*000 people died unnecessarily in South Africa over the period and that 35*,*000 HIV-infected babies were born that could have been protected from the virus and would probably have a limited life *[4]*.*

*This lack of decisive South African government action in the face of immense human suffering and unnecessary loss of lives led to huge civil society outrage. AIDS activists from a previously little known non-profit group called the Treatment Action Campaign* (*TAC*), *assisted by the AIDS Law Clinic from the University of the Witwatersrand’s Centre for Applied Legal Studies* (*CALS*), *stepped into the breach and forced the government to honor the legal duty of care – owed to South African citizens living with HIV/AIDS. Political advocacy by the TAC garnered much international support for the cause. Under international pressure*, *South Africa eventually launched a national program for the prevention of mother-to-child-transmission in August 2003 and a national adult treatment program in 2004 *[4]*. The TAC’s lobby efforts were also directed against pharmaceutical multinational companies whose high drug prices and strangulate drug patents made access to life-saving medicines for patients difficult*, *especially the poor. “Expensive antiretrovirals* (*ARVs*) *came down in price dramatically as a result of activists’* (*political advocates’*) *campaigning and public pressure.” *[4]*.*

South Africa’s national HIV/AIDS treatment-care landscape has changed dramatically since 2004 through the action of TAC. On December 1, 2011 (World AIDS Day), President Jacob Zuma announced a plan to fight against HIV/AIDS. The advocacy of the TAC in the 1990s to the mid-2000s chartered and eased the path for effective collaboration between government and HIV/AIDS patient groups. Great progress has been attained in how key HIV/AIDS healthcare policies are developed and implemented in South Africa. Government and healthcare policymakers now appreciate the value of civil society engagement as key stakeholders in healthcare planning and implementation.

Cancer advocates and lobby groups would do well to take critical lessons from their HIV/AIDS counterparts and find strategies to engage the government in similar interactions to facilitate the development of a focused, sustainable and scalable National Strategic Plan for Cancer grounded in evidence based research. Bruising battles have been fought and won at grave human cost and there is no need to repeat painful mistakes – and to cause similar unnecessary deaths and suffering for cancer patients and their families. For example, the comprehensive multi-disciplinary and multi-sectoral HIV/AIDS approach, introduced by President Zuma in South Africa, presents a prime opportunity for partnerships between national HIV/AIDS and cancer political advocates. The two diseases sometimes occur as co-morbidities in the same patient and it would therefore be highly cost effective to develop and implement integrated research and treatment plans. Sufficient research exists on the high incidence of AIDS defining carcinomas, like Kaposi sarcoma, which is one of the four common cancers in both men and women in sub-Saharan Africa. There are also biological connections between HIV/AIDS and tuberculosis, cervical and breast cancers among African patients. A strategic approach to cancer control (factoring these listed diseases) in sub-Saharan Africa is needed to build on what works and what is unique to the region.

## How science educated patient advocates can drive cancer advocacy and influence research and political agendas: Lessons from Project LEAD Program, United States

*“They are not scientists but their connection to cancer is no less strong”*,[5] writes Kana about the Georgetown Lombardi’s Patient Advocacy Committee (GLBCPAC), a non-profit organization formed by Ayesha Shajahan-Haq. “*Encouraging researchers*, *basic or clinical*, *to work with advocates is the right step to help in bridging the gap between cancer research and the community it is intended to directly impact”* says Shajahan-Haq.[5] The GLBCPAC is made up of 14 breast cancer patient advocates and five of them are formally trained National Breast Cancer Coalition Project LEAD graduates [5].

Project LEAD is the National Breast Cancer Coalition’s (NBCC) premier science training program for patient advocates in the United States and has created a revolution in the world of breast cancer research and public policy. The courses prepare graduates to engage in a wide range of local and national forums where breast cancer decisions are made. Project LEAD graduates bring an educated consumer perspective and critical thinking skills to the important issues and controversies in breast cancer [6].

As a result of NBCC’s work, scientists, government agencies and private industry have changed the way they design and implement breast cancer research and programs. NBCC has created a model for consumer influence marked by transparency, innovation and a peer relationship among scientists, researchers, policymakers and consumers [6] in the United States. In addition, Project LEAD International is slowly growing this influence globally. The respect for and influence of NBCC Project LEAD graduates is not limited to research science but also extends to high level political structures. The NBCC has had tremendous success in influencing United States national public-policy on breast cancer research. For example, the Department of Defense (DOD) Breast Cancer Research Program (BCRP) was created in 1992 as a result of the organization’s campaign to increase United States federal funding for breast cancer research [7]. Strategic partnership with supportive Congressional leaders like Senators Tom Harkin (a democrat from Iowa) and Alfonse D’ Amato (republican from New York) led the United States Congress to appropriate $210 million in the DOD research and development budget for breast cancer peer-reviewed research program administered by the United States Department of the Army, during the 1993 financial year [7]. As a result of Senator Harkin’s continued support, NBCC grassroots advocacy, and the DOD BCRP’s demonstrated success, Congress has approved funding for breast cancer research annually [7].

It is worth noting that the development of the drug Herceptin which has saved the lives of many women with HER-2 positive breast cancer is one of the key successes of the DOD BCRP. United States Speaker Nancy Pelosi’s 2010 NBCC Congressional Awards acceptance speech when she was presented with the Public Policy Leadership Award, best capture the high political regard accorded the patient advocate organization on Capitol Hill:

*“I know the National Breast Cancer Coalition’s number one legislative priority is access to quality health care for all. And because of your advocacy*, *your organization*, *your number one legislative priority is now the law of the land.” *[8]*. Already we are seeing progress. As you know*, *the media has reported that certain insurance companies were particularly targeting women with breast cancer diagnoses for rescission. But because of your leadership*, *America largest insurers acted even sooner than the health insurance reform bill we passed required to end the shameful practice of dropping women’s coverage when they get sick”.*[8]*We are counting on* (*the NBCC*) *to continue to work with us to educate the American people about what health care reform means to them” *[8]*. I know you have come to Capitol Hill to lobby for increased funding for breast cancer research. We have been working together on this issue for years. In the early years*, *our battle was to make sure the NIH was directing resources to breast cancer. I remember when we broke the first $100 million mark for breast cancer funding. With the help of Jack Murtha*, *together we created the Breast Cancer Research Program at the Defense Department*, *which has invested $2 billion in research since 1993.” *[8]*.*

Between 1992 and 2012, the power of political breast cancer advocacy resulted in $2.78 billion United States Congregational appropriation for the DOD Breast Cancer Research Program and over 6,000 grant awards. The same success can be achieved in Africa with the right partnership between the government and advocates.

## Conclusion

Patient advocates have carved themselves a niche and earned trust as peers and key partners in research, clinical and policymaking forums. Although the patient advocacy movement is still relatively young in Africa compared to Western countries like the United States, the South African Treatment Action Campaign and its patient alliance partners showed their mettle in the formidable battle to secure equitable access to evidence-based HIV/AIDS treatment to millions of people who needed it. Great strides in patient engagement have been made by South African healthcare policymakers. The Minister of Health showed a strong commitment to patient inclusion by appointing three lay persons to his inaugural Cancer Advisory Committee – which started its 3-year term in April 2013. A patient has also been elected to serve as Deputy Chair of the Committee.

There are great lessons and partnerships to be forged between HIV/AIDS and cancer patient advocates. In addition, broader synergies and partnerships need to be fostered among clinicians, researchers and policymakers to create integrated and context relevant, cost effective and sustainable healthcare systems in Africa. Lessons from the United States based NBCC Project LEAD and the GLBCPAC also serve as great illustrations of the impact and value that science trained patient advocates can bring to both the research and policymaking arenas. Testimonies from clinicians, researchers and high level public office holders offer evidence of how patient inclusion in key healthcare forums is a public policy, societal and business imperative. The examples offer lessons for African countries to emulate.

The author is a a Project LEAD graduate of the NBCC Project LEAD, a member of the NBCC and Deputy Chair of the South Africa Minister of Health Cancer Advisory Committee.

## Abbreviations

AIDS: Acquired Immunodeficiency Syndrome; ARV: Antiretroviral; BCRP: Breast Cancer Program; CALS: Centre for Applied Legal Studies; DOD: Department of Defense; GLBCPAC: Georgetown Lombardi’s Patient Advocacy Committee; HIV: Human Immunodeficiency Virus; NBCC: National Breast Cancer Coalition; NGOs: Non-governmental Organizations; TAC: Treatment Action Campaign; US: United States; WHO: World Health Organization.

## Competing interests

The author declares to have no competing interests.

## Declarations

The authors declare that funding for publication of the article was funded by an award provided by the National Cancer Institute (Award Number R13CA162899).

This article has been published as part of *Infectious Agents and Cancer* Volume 8 Supplement 1, 2013: Selected articles from The International Workshop on Cancer Advocacy for African Countries (CAAC) 2011. The full contents of the supplement are available online at http://www.infectagentscancer.com/supplements/8/S1 The Supplement Editors declare they have no competing interests.
